# Synchronous Neoplasia Rates at Colonoscopic Diagnosis of Early-Onset vs Average-Onset Colorectal Cancer

**DOI:** 10.1001/jamanetworkopen.2023.24038

**Published:** 2023-07-18

**Authors:** Oluwadunni E. Emiloju, Bahar Saberzadeh-Ardestani, Frank A. Sinicrope

**Affiliations:** 1 Department of Medicine, Mayo Clinic and Mayo Alix School of Medicine, Rochester, Minnesota; 2 Department Oncology, Mayo Clinic and Mayo Alix School of Medicine, Rochester, Minnesota; 3 Mayo Clinic Comprehensive Cancer Center, Rochester, Minnesota

## Abstract

**Importance:**

The incidence of early-onset colorectal cancer (CRC) (age, <50 years) continues to increase globally within high-income countries.

**Objective:**

To examine and compare rates of synchronous neoplasia found in patients at colonoscopic diagnosis of early-onset CRC with rates found at diagnosis of average-onset CRC.

**Design, Setting, and Participants:**

In this multisite retrospective and cross-sectional study conducted at Mayo Clinic sites and in the Mayo Clinic Health System from January 1, 2012, to December 31, 2022, 150 randomly selected patients with early-onset CRC were identified from the electronic health record and matched with 150 patients with average-onset CRC based on sex and colonoscopic indication. Patients with known hereditary syndromes, past history of CRC, or inflammatory bowel disease were excluded.

**Main Outcomes and Measures:**

Colonoscopic findings (polyp size, number, site) and related histopathologic findings (adenoma, advanced adenoma, sessile serrated polyp) were analyzed in association with cancer clinicopathologic features and molecular data (mismatch repair status, *KRAS*, and *BRAFV600E*).

**Results:**

Among 300 patients (156 men [52%]), the median age at diagnosis was 43 years (IQR, 39-47 years) for those with early-onset CRC and 67 years (IQR, 57-76) for those with average-onset CRC. Overall, 85% of patients were symptomatic at CRC diagnosis. Cancer stage, grade, molecular features, body mass index, and family history did not differ significantly between these groups. Among patients with colon cancer, the overall prevalence of synchronous neoplasia was similar, yet advanced adenomas were 3 times more frequent in those with early-onset vs average-onset cancers (31 of 75 [41%] vs 10 of 75 [13%]; *P* < .001). This difference was not associated with cancer stage or primary location. Among patients with rectal cancer, nonadvanced adenomas were less frequent among the early-onset group than the average-onset group (21 of 75 [28%] vs 36 of 75 [48%]), and although the prevalence of advanced adenomas was similar (11 of 75 [15%] vs 14 of 75 [19%]), they were more commonly located in the rectum (early onset, 5 of 11 [45%] vs average onset, 1 of 14 [7%]). Patients with early-onset cancer of the colon were significantly more likely than those with early-onset cancer of the rectum to have a synchronous advanced adenoma (31 of 75 [41%] vs 11 of 75 [15%]; *P* < .001).

**Conclusions and Relevance:**

In this cross-sectional study, synchronous advanced adenomas were more commonly found in patients with early-onset colon cancer compared with average-onset colon cancer, and they were distributed throughout the colon. In contrast, advanced adenomas were not increased in patients with rectal cancer and, when detected, were predominantly located in the rectum.

## Key Points

**Question:**

At colonoscopic diagnosis of colorectal cancer, do patients with early-onset vs average-onset of disease have different rates of synchronous colorectal neoplasia?

**Findings:**

In this cross-sectional study of 300 patients, patients with early-onset colon cancer, but not rectal cancer, had significantly higher rates of synchronous advanced adenomas compared with patients with average-onset cancers.

**Meaning:**

The finding of increased synchronous advanced adenomas among patients with early-onset colon cancer, but not rectal cancer, suggests widespread field cancerization in the former, indicating biological differences by primary tumor site.

## Teaser

This cross-sectional study examines and compare the rates of synchronous neoplasia found at colonoscopy among patients with early-onset colorectal cancer (CRC) with the rates found among patients with average-onset CRC.

## Introduction

Colorectal cancer (CRC) remains the third most common cancer and is the third leading cause of cancer-related mortality in the United States.^1^ Although the incidence of CRC continues to decrease among adults older than 50 years, likely due to population-based screening with colonoscopy,^1,2,3,4^ the incidence among adults younger than 50 years has steadily increased by 2% annually since the 1990s.^5^ Early-onset CRC, defined as diagnosis at an age younger than 50 years, currently accounts for 10% of all cases of CRCs^5,6^ and is estimated to account for 1 in 10 cases of colon cancer and 1 in 4 cases of rectal cancer by 2030.^7^ Primary tumors in early-onset CRC are located predominantly in the distal colon and rectum compared with average-onset CRC,^8,9,10^ with rectal cancer being most frequent in the US.^8,9,10^ Patients with early-onset CRC often experience a delay in diagnosis,^9,11^ which may account for their more advanced stage at presentation, although a more aggressive biology is suggested.^9^ Most cases of early-onset CRC are sporadic, and a prospective study of early-onset CRCs identified germline variants in 16% of patients, of whom approximately one-half had Lynch syndrome.^12^

Although synchronous adenomas are often present at diagnosis among patients with CRC, sparse data exist for patients with early-onset CRC.^13,14,15^ Such data can provide insight into field cancerization^16^ and risk of metachronous neoplasia.^17^ In a study of 1522 patients with CRC, synchronous colorectal neoplasms were observed in 505 patients (33%).^13,14^ Colonoscopy enables the removal of established CRC precursor lesions, including adenomas, advanced adenomas, and sessile serrated lesions.^18^ Patients with an advanced adenoma had a 2.5-fold greater risk of subsequent development of CRC compared with those without an adenoma.^19^ Serrated lesions can progress through the serrated neoplasia pathway characterized by a variant in *BRAF* (V600E) (OMIM 164757), disruptions in *Wnt* signaling, and widespread CpG island methylation.^20,21^

We examined synchronous neoplasia at colonoscopic diagnosis of early-onset CRC compared with average-onset CRC given the potential insights into field cancerization and risk of metachronous CRC. We sought to test the hypothesis that patients with early-onset CRC may have lower rates of synchronous neoplasia given that adenoma prevalence increases substantially with age.^22,23^ We also examined synchronous neoplasia in association with primary cancer location (ie, colon vs rectum) given differences in their biology, which may affect findings at colonoscopy.^8,9^

## Methods

Patients with primary early-onset colon and rectal adenocarcinomas (age at diagnosis, 18-49 years) seen at Mayo Clinic sites (Rochester, Minnesota; Jacksonville, Florida; and Scottsdale, Arizona) or the Mayo Health System from January 1, 2012, to December 31, 2022, were identified from the electronic health record (EHR) using Epic Slicer Dicer. The patient list was exported, random numbers were generated and assigned for each case, and cases were sorted numerically using Excel software, version 2021 (Microsoft Corp). Each patient’s EHR was then reviewed based on strict inclusion and exclusion criteria until we met the projected sample size (see Statistical Analysis). Eligible patients had optical (high-definition white light) colonoscopy to the cecum with at least fair bowel preparation, with diagnosis of CRC confirmed by histopathologic findings. Exclusion criteria included patients with inflammatory bowel disease, known familial adenomatous polyposis or Lynch syndrome, 10 or more polyps at index colonoscopy, or prior CRC. A total of 150 patients with early-onset CRC were matched with patients with average-onset colon cancer (n = 75) and rectal cancer (n = 75) based on sex and indication for colonoscopy because these factors are known to influence colonoscopic findings.^24,25^ Matching cases for patients with early-onset CRC were identified by consecutive review of a list of randomly selected patients with average-onset CRC until an equivalent number of matched cases was obtained. The relative proportion of early-onset to average-onset CRC cases was similar across study sites. This study was reviewed and approved by the Mayo Clinic institutional review board, which waived patient consent because this was a retrospective analysis of deidentified data. The study follows the Strengthening the Reporting of Observational Studies in Epidemiology (STROBE) reporting guideline for observational studies.

Data on synchronous neoplasia size, number, and location in the colorectum were abstracted from colonoscopy and related pathology reports that recorded histopathologic interpretation of biopsy or polypectomy specimens. Synchronous neoplasia included adenomas, advanced adenomas (≥1 cm, villous histologic characteristics, and/or high-grade dysplasia) and sessile serrated polyps. Hyperplastic polyps were excluded from the study analysis given their lack of neoplastic potential.^21^ Data were collected for DNA mismatch repair (MMR) status (deficient [dMMR] vs proficient [pMMR]) and *BRAF*^V600E^ or *KRAS* (OMIM 190070; variant vs wild type [WT]) on primary CRCs. Where available, results of multigene germline testing data were also abstracted, and germline testing had been performed for patients with dMMR early-onset CRC to exclude Lynch syndrome.

Clinicopathologic variables, including sex (female vs male), primary tumor site (right [proximal to the splenic flexure] vs left), tumor grade (G3 vs G1/2), and TNM disease stage (I-IV), were recorded. Body mass index (BMI) and family history of CRC (first- and second-degree relatives) were abstracted from the EHR. Symptoms at diagnosis of CRC were also recorded.

### Statistical Analysis

Data on synchronous neoplasia number, size, site, and histopathologic characteristics were compared between patients with early-onset colon cancer and patients with early-onset rectal cancer and with their respective matched cohorts with average-onset CRC. Based on our study hypothesis, we assessed and compared rates of synchronous neoplasia between groups. Sample size calculations indicated that 75 patients per group (total, 150) provided 80% power to detect a 20% difference in the rate of synchronous neoplasia between patients with early-onset CRC and patients with average-onset CRC at a 2-sided α of .10, which we regarded as a clinically meaningful difference. Our estimate for prevalence of synchronous adenomas (33%) was based on the literature.^14^ Continuous variables are described as median (IQR) values and were compared using the Mann-Whitney test. The Fisher exact test was used to compare categorical variables exclusively due to sample size. Two-sided *P* values are reported, and *P* < .05 was considered statistically significant for all analyses. Statistical analysis was performed with R studio software, version 2022.02.3 (R Group for Statistical Computing).

## Results

### Colon Cancer

A total of 72 patients with colon cancer were male (48%) and 78 (52%) were female. The median age of patients with early-onset colon cancer (n = 75) was 45 years (range, 28-49 years), and the median age was 71 years (range, 50-92 years) for the randomly selected, matched cohort of patients with average-onset colon cancer (n = 75). Indications for colonoscopy were diagnostic, surveillance, and screening in 79% (n = 118), 11% (n = 16), and 11% (n = 16) of cases, respectively. Among patients with colon cancer, 118 (79%) exhibited symptoms at diagnosis. Among patients with early-onset colon cancer, gastrointestinal bleeding (24 of 59 [41%]), mostly hematochezia, followed by abdominal pain (18 of 59 [31%]) were the most common symptoms at cancer diagnosis, and gastrointestinal bleeding was significantly more common compared with patients with average-onset colon cancer (Table 1). Primary cancers in the left colon were more common among patients with early-onset colon cancer compared with those with average-onset colon cancer (38 [51%] vs 24 [32%]; *P* = .02). Body mass index was similar between patients with early-onset and average-onset colon cancer (*P* = .15).

**Table 1.** Clinicopathologic Characteristics of Patients at Diagnosis of Early-Onset vs Average-Onset Colon and Rectal Cancer

Characteristic^a^	Colon cancer		*P* value	Rectal cancer		*P* value
	Early onset (n = 75)	Average onset (n = 75)		Early onset (n = 75)	Average onset (n = 75)	
Age at diagnosis, median (IQR), y	45 (40-47)	71 (64-80)	NA	43 (39-46)	60 (55-70)	NA
Family history of colon cancer, No. (%)						
Yes	23/72 (32)	22/75 (29)	.85^b^	24/74 (32)	24/74 (32)	>.99^b^
No	49/72 (68)	53/75 (71)		50/74 (68)	50/74 (68)	
Symptoms at presentation						
Abdominal pain	18/59 (31)	13/59 (22)	.04^b^	7/68 (10)	4/68 (6)	.61^b^
Anemia	7/59 (12)	17/59 (29)		0	2/68 (3)	
Gastrointestinal bleeding	24/59 (41)	13/59 (22)		53/68 (78)	56/68 (82)	
Incidental finding on imaging	6/59 (10)	7/59 (12)		1/68 (2)	1/68 (2)	
Other	4/59 (6)	9/59 (15)		7/68 (10)^c^	5/68 (7)	
Primary tumor site, No. (%)						
Left	38/74 (51)	24/75 (32)	.02^b^	NA	NA	NA
Right	36/74 (49)	51/75 (68)		NA	NA	
Histologic grade, No. (%)						
G1/2	56/67 (84)	57/66 (86)	.81^b^	54/69 (78)	56/64 (88)	.18^b^
G3	11/67 (16)	9/66 (14)		15/69 (22)	8/64 (12)	
TNM stage, No. (%)						
I	14/73 (19)	14/72 (19)	>.99^b^	10/73 (14)	11/74 (15)	.20^b^
II	14/73 (19)	15/72 (21)		4/73 (6)	11/74 (15)	
III	23/73 (32)	22/72 (31)		32/73 (44)	33/74 (45)	
IV	22/73 (30)	21/72 (29)		27/73 (37)	19/74 (26)	
MMR status, No. (%)						
dMMR	3/68 (4)^d^	9/64 (14)	.07^b^	2/69 (3)^e^	1/69 (1)	>.99^b^
pMMR	65/68 (96)	55/64 (86)		67/69 (97)	68/69 (99)	
*KRAS*, No. (%)						
Variant	14/29 (48)	21/40 (52)	.81^b^	17/43 (40)	16/35 (46)	.65^b^
WT	15/29 (52)	19/40 (48)		26/43 (60)	19/35 (54)	
*BRAF*, No. (%)						
Variant (V600E)	2/32 (6)	6/40 (15)	.29^b^	2/45 (4)	1/34 (3)	>.99^b^
WT	30/32 (94)	34/40 (85)		43/45 (96)	33/34 (97)	
BMI at diagnosis, median (IQR)	26 (23-30)	24 (23-28)	.15^f^	28 (23-33)	29 (26-33)	.37^f^

Abbreviations: BMI, body mass index (calculated as weight in kilograms divided by height in meters squared); dMMR, deficient MMR; MMR, mismatch repair; NA, not applicable; pMMR, proficient MMR; TNM, tumor, node, metastasis; WT, wild type.

^a^ Sum of the numbers from each subcategory may not equal the total reported cases due to missing values. Missing values are not included in the percentages.

^b^ Fisher exact test.

^c^ Included 3 patients presenting with alteration in bowel habits.

^d^ One patient with negative germline test and 2 patients with *MLH1* hypermethylation.

^e^ One patient with negative germline test, other patient with negative family history, *BRAF* status unknown.

^f^ Mann-Whitney test.

At cancer diagnosis, TNM stage and tumor molecular markers (MMR, *BRAF*^V600E^, or *KRAS*) did not differ significantly between patients with early-onset and patients with average-onset colon cancer (Table 1). No differences were found for patient-reported family history of CRC in our study population in which patients with familial adenomatous polyposis or Lynch Syndrome had been excluded. Among patients who had germline genetic testing, such testing was more frequent for patients with early-onset vs average-onset colon cancer (41 [55%] vs 15 patients [20%]). Among patients with average-onset colon cancer, 7 of 15 patients had germline data that were generated in conjunction with somatic tumor next-generation sequencing profiling by TEMPUS; the other 8 cases were tested due to family history of cancer. At least 1 pathogenic germline variant (PGV) was identified for 6 of 41 tested patients with early-onset colon cancer (15%) and for 1 of 15 tested patients with average-onset colon cancer (7%) (Box). Among the PGVs identified in patients with early-onset colon cancer, *CHEK2* (OMIM 604373) found in 1 patient was the only PGV with a known yet weak association with risk of CRC.^26^

Box. **Cases of Early-Onset Colon and Rectal Cancer With Finding of a Germline Pathogenic Variant**

**Early-Onset Colon Cancer^a^**

- *ATM*
- *CHEK2*
- *FH*
- *MUTYH* (monoallelic)
- *BRCA1*
- *EPCAM* (exon 5 deletion, monoallelic)

**Early-Onset Rectal Cancer^a^**

- *CHEK2, RAD50*
- *CHEK2*
- *MUTYH, RECQL4* (both monoallelic)

^a^
Each line represents an individual patient.

**Figure 1.**
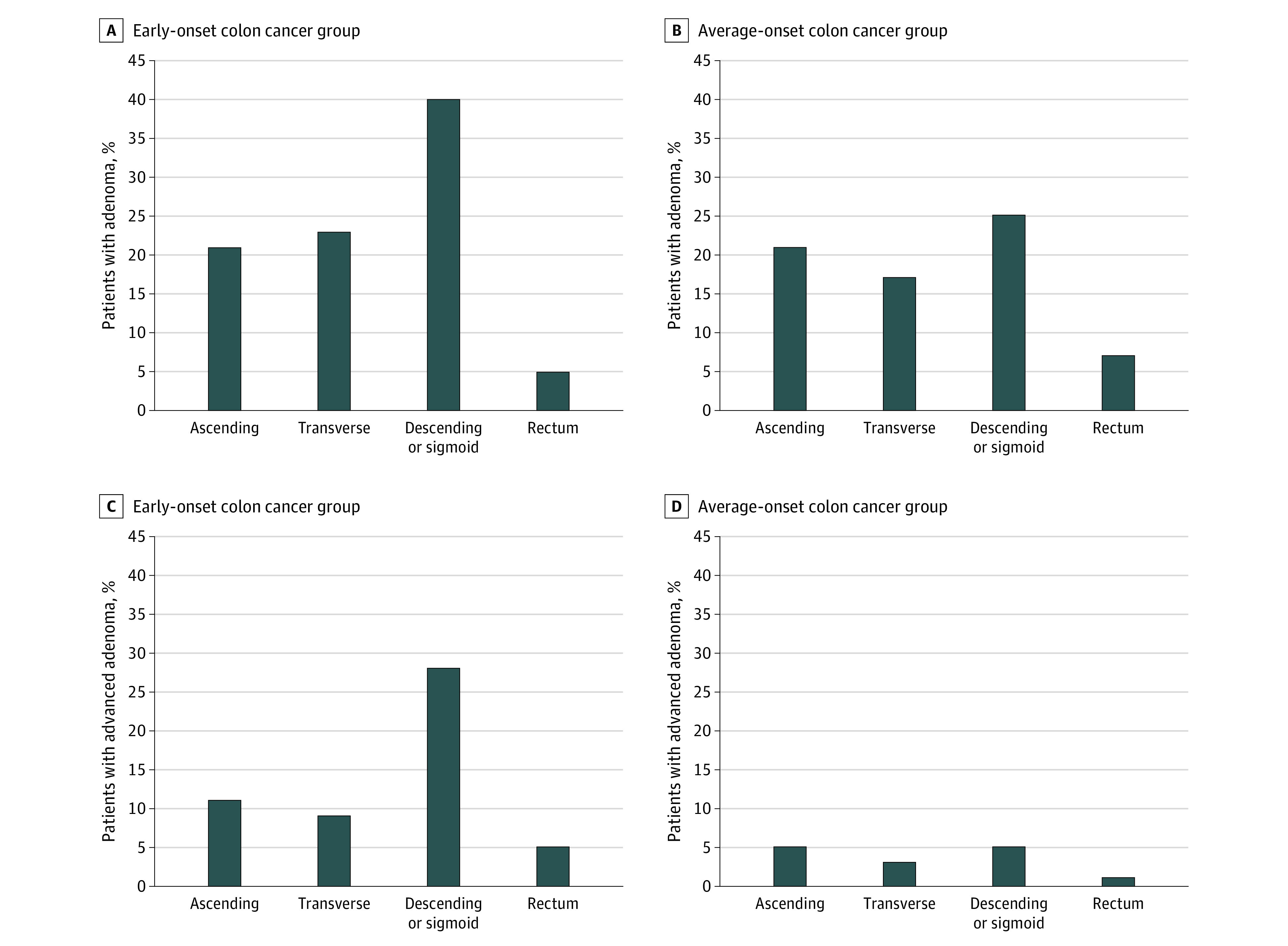
Patients With Early-Onset or Average-Onset Colon Cancer and Adenoma or Advanced Adenoma Among patients with early-onset or average-onset colon cancer, the percentages of those with adenoma (A and B) or advanced adenoma (C and D) by site within the colorectum are shown. In contrast to adenomas (*P* = .33), advanced adenomas were significantly more common among patients with early-onset vs average-onset colon cancer (41% vs 13%; *P* < .001).

At colonoscopic diagnosis of colon cancer, at least 1 synchronous adenoma was found in 42 patients with early-onset disease (56%) and 35 patients with average-onset disease (47%) (Table 2). The median adenoma size was significantly larger in patients with early-onset-colon cancer compared with those with average-onset colon cancer (10 mm [IQR, 7-14 mm] vs 5 mm [IQR, 4-7 mm]; *P* = .001) (Table 2). Advanced adenomas were significantly more common in the early-onset colon cancer group compared with the average-onset colon cancer group (31 of 75 [41%] vs 10 of 75 [13%]; *P* < .001), and they were more common in the left colon (Figure 1), which was maintained when the cohort was limited to pMMR tumors (*P* = .001). The likelihood of synchronous advanced adenomas was not associated with colon cancer stage at diagnosis, *BRAF* status, or primary tumor location. A total of 30 of 118 patients with colon cancer with symptoms (25%) had advanced adenoma. Of the 31 patients with early-onset colon cancer and advanced adenoma, 17 had germline testing, and 3 patients had PGVs (*BRCA1*, monoallelic *EPCAM* [OMIM 185535], and monoallelic *MUTYH* [OMIM 604933]). Among the 10 patients with average-onset colon cancer and advanced adenoma, 3 had germline testing, and 1 PGV was found in *MUTYH* (monoallelic). The prevalence of sessile serrated polyps was similar between patients with early-onset colon cancer and patients with average-onset colon cancer (Table 2). Among patients with early-onset colon cancer, adenomas with high-grade dysplasia were found in 15 patients (20%) compared with 0% for patients with average-onset colon cancer.

**Table 2.** Characteristics of Synchronous Polyps at Diagnosis of Colon and Rectal Cancer: Early Onset vs Average Onset

Characteristic	Colon cancer		*P* value	Rectal cancer		*P* value
	Early onset (n = 75)	Average onset (n = 75)		Early onset (n = 75)	Average onset (n = 75)	
All adenomas						
Detected, No. (%)	42 (56)	35 (47)	.33^a^	21 (28)	36 (48)	.02^a^
No. per patient, median (IQR)	2 (1-3)	2 (1-3)	.35^b^	1 (1-2)	2 (1-3)	.15^b^
Size, median (IQR), mm	10 (7-14)	5 (4-7)	.001^b^	7 (4-10)	5 (4-9)	.48^b^
Right, No. (%)	27 (64)	28 (78)	.22^a^	8 (38)	27 (75)	.01^a^
Left, No. (%)	31 (74)	22 (59)	.23^a^	14 (67)	19 (53)	.41^a^
Advanced adenoma						
Detected, No. (%)	31 (41)	10 (13)	<.001^a^	11 (15)	14 (19)	.66^a^
No. per patient, median (IQR)	1 (1-2)	2 (1-2)	.34^b^	1 (1-1)	1 (1-1)	.69^b^
Size >10 mm, No. (%)	22 (31)	9 (12)	.005^a^	10 (14)	10 (14)	>.99^a^
Tubullovillous or villous, No. (%)	19 (25)	4 (5)	.001^a^	3 (4)	5 (7)	.72^a^
High dysplasia, No. (%)	15 (20)	0	<.001^a^	2 (3)	3 (4)	>.99^a^
Right, No. (%)	14/31 (45)	5/10 (50)	>.99^a^	2/11 (18)	7/14 (50)	.21^a^
Left, No. (%)	24/31 (77)	5/10 (50)	.12^a^	9/11 (82)	7/14 (50)	.21^a^
Sessile serrated polyp						
Detected, No. (%)	8 (11)	10 (14)	.62^a^	8 (11)	5 (7)	.56^a^
No. per patient, median (IQR)	1 (1-1)	1 (1-1)	.85^b^	1 (1-1)	3 (2-3)	.005^b^
Size, median (IQR), mm	5 (4-8)	5 (3-11)	.86^b^	5 (4-11)	4 (4-6)	.51^b^
High dysplasia, No. (%)	0	0	>.99^a^	0	0	>.99^a^
Right, No. (%)	4/8 (50)	5/10 (50)	>.99^a^	6/8 (75)	2/5 (40)	.29^a^
Left, No. (%)	5/8 (62)	6 /10(60)	>.99^a^	2/8 (25)	3/5 (60)	.29^a^

^a^ Fisher exact test.

^b^ Mann-Whitney test.

### Rectal Cancer

A total of 84 patients with rectal cancer were male (56%) and 66 (44%) were female. The median age at cancer diagnosis of patients with early-onset rectal cancer (n = 75) was 43 years (range, 26-49 years), and the median age was 60 years (range, 50-85 years) for the randomly selected matched cohort of patients with average-onset rectal cancer (n = 75) (Table 1). Indications for colonoscopy were diagnostic for 91% (n = 136), screening for 8% (n = 12), and surveillance for 1% (n = 2) of cases. Among patients with rectal cancer, 136 (91%) exhibited symptoms at diagnosis. The most common symptoms at diagnosis of early-onset and average-onset rectal cancer were hematochezia (53 of 68 [78%] vs 56 of 68 [82%]), followed by abdominopelvic pain and alteration in bowel habits (Table 1). TNM stage and molecular markers (MMR, *BRAF*^V600E^, and *KRAS*), BMI, and family history were similar between patients with early-onset rectal cancer and patients with average-onset rectal cancer (Table 1). Among patients for whom germline data were available, testing was more frequently performed for patients with early-onset vs average-onset rectal cancer (36 [48%] vs 19 [25%]). Reasons for genetic testing among 19 patients with average-onset rectal cancer included 5 for whom germline data were included with somatic tumor next-generation sequencing, 4 due to enrollment in research studies, and the remainder prompted by family history of cancer. Three of 36 tested patients with early-onset rectal cancer (8%) had a PGV, and 1 of 19 tested patients with average-onset rectal cancer (5%) had a PGV (Box).

**Figure 2.**
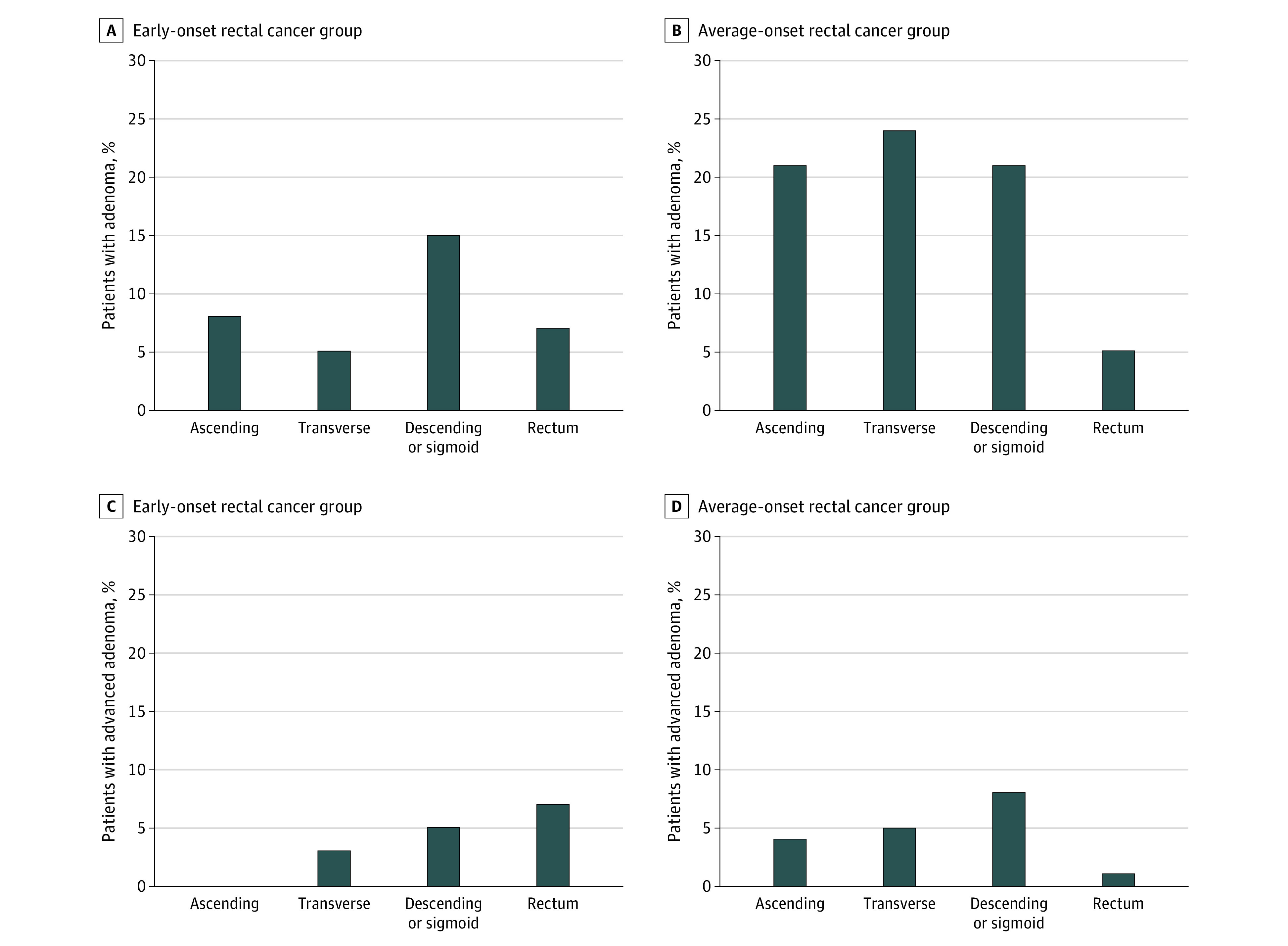
Patients With Early-Onset or Average-Onset Rectal Cancer and Adenoma or Advanced Adenoma Among patients with early-onset or average-onset rectal cancer, the percentages of those with adenoma (A and B) or advanced adenoma (C and D) by site within the colorectum are shown. Although adenoma distribution was similar by age group, advanced adenomas were significantly more common in the rectum among early-onset compared with average-onset rectal cancers (45% vs 7%; *P* < .001).

At colonoscopic diagnosis of rectal cancer, adenomas were significantly less common in the early-onset rectal cancer group compared with the average-onset rectal cancer group (21 of 75 [28%] vs 36 of 75 [48%]; *P* = .02) (Table 2); however, the prevalence of advanced adenomas was similar between these 2 groups (11 of 75 [15%] vs 14 of 75 [19%]). Advanced adenomas were significantly more common in the rectum among patients with early-onset vs average-onset rectal cancer (early-onset, 5 of 11 [45%] vs average-onset, 1 of 14 [7%]; *P* < .001) (Figure 2). Of the 11 patients with early-onset rectal cancer with advanced adenoma, 4 had germline testing, and no PGVs were found. Similarly, 5 of 14 patients with average-onset rectal cancer with advanced adenoma had germline testing, and no PGVs were identified. A total of 21 of 136 symptomatic patients with rectal cancer (15%) had advanced adenoma. The frequency of sessile serrated polyps did not differ significantly between these groups (Table 2).

### Colon vs Rectal Cancer

The prevalence of adenomas was significantly increased among patients with early-onset colon cancer compared with those with early-onset rectal cancer (42 [56%] vs 21 [28%]; *P* < .001) yet did not differ between these groups for patients with average-onset colon cancer and rectal cancer (35 [47%] vs 36 [48%]). For patients with early-onset but not average-onset cancer, those with colon vs rectal cancers were significantly more likely to have synchronous advanced adenomas (early onset, 31 of 75 [41%] vs 11 of 75 [15%]; *P* < .001; average onset, 10 of 75 [13%] vs 14 of 75 [19%]; *P* = .50).

## Discussion

We performed the first study, to our knowledge, to compare synchronous neoplasia at colonoscopic diagnosis of early-onset vs average-onset colon and rectal cancers. We hypothesized that patients with early-onset CRC may have fewer synchronous neoplasms. Among patients with colon cancer, we found that the overall number of adenomas and their left colon predominance were similar for both age groups. However, there was a 3-fold increase (41% vs 13%) in advanced adenomas among patients with early-onset compared with average-onset colon cancer whose distribution favored the left colon; this increase was not associated with cancer stage, clinicopathologic variables, or limited molecular markers. Of note, 85% of patients in the overall cohort were symptomatic at diagnosis of CRC. Among patients with early-onset colon cancer, adenomas with high-grade dysplasia were found in 20% of patients compared with 0% of patients with average-onset colon cancer. These data suggest accelerated carcinogenesis and extended field cancerization in early-onset colon cancer cases. Of 31 patients with early-onset colon cancer found to have advanced adenomas, 17 had germline testing data, and 3 patients had PGVs (1 each in *BRCA1*, *EPCAM* [monoallelic], and *MUTYH* [monoallelic] genes). The observed exon 5 deletion in *EPCAM* has not been associated with epigenetic silencing of *MSH2*, and the monoallelic *MUTYH* variant is associated with only a modest increase in CRC risk.^27^ Among the 10 patients with average-onset colon cancer and advanced adenomas, 3 had germline testing data, and 1 had a monoallelic *MUTYH* variant. Although germline data were available for only a subset of patients, such data do not suggest an explanation for the increase in advanced adenomas among patients with early-onset vs average-onset colon cancer.

Colon and rectal cancers were analyzed separately given their purported biological differences and the fact that most cases of early-onset cancers arise in the rectum in the US population.^8,9,10^ In contrast to patients with early-onset colon cancer, those with early-onset vs average-onset rectal cancer had fewer nonadvanced adenomas but similar numbers of advanced adenomas that were more commonly found in the distal colorectum. Among patients with early-onset rectal cancer and advanced adenoma, 4 of 11 had germline testing, and no PGVs were found, as was also the case for 5 of 14 patients with average-onset rectal cancer and advanced adenomas. Comparing patients with early-onset colon cancer vs rectal cancer revealed that only patients with colon cancer were significantly more likely to have synchronous advanced adenomas. Our finding of high rates of synchronous colorectal neoplasia indicates the necessity to perform complete colonoscopy when possible, at diagnosis of early-onset CRC, or after surgery in cases with obstruction. Increased advanced adenomas among patients with colon cancer, but not among patients with rectal cancer, underscores an elevated risk of synchronous CRC.^28^

Regarding risk factors for CRC, the number of first- and second-degree relatives with CRC did not differ significantly between patients with early-onset vs average-onset CRC. Although obesity appears to be a risk factor for development of CRC,^29,30,31^ BMI^32,33,34^ did not differ between patients with early-onset CRC and patients with average-onset CRC in our study. In tumor tissue, *KRAS* and *BRAF*^V600E^ variant frequencies were similar for patients with early-onset CRC and patients with average-onset CRC, which is consistent with results for next-generation sequencing in a consecutive series of 759 patients with early-onset vs average-onset CRCs, in which their somatic tumor genomes were indistinguishable.^8^

Field cancerization describes a field of cellular and molecular alterations that predispose the individual to the development of colorectal neoplasia.^35^ We observed increased synchronous neoplasia throughout the colorectum in patients with early-onset colon cancers, suggestive of extended field cancerization whereby events involved in carcinogenesis occur in normal-appearing tissue away from a cancer that can progress to precursor lesions.^16^ Our data suggest that such field cancerization may be more limited among patients with early-onset rectal cancer, for whom the distribution of adenomas was more limited to the distal colorectum. Multiple etiologic factors, including dietary, lifestyle, microbial, and genetic variables, exert their influence and interactions to contribute to field cancerization and susceptibility to neoplastic development and progression. Future research in early-onset CRC is needed to identify endogenous and environmental exposures that can influence the genome, epigenome, transcriptome, proteome, and microbiome or metabolome in addition to host immunity.

### Limitations and Strengths

This study has some limitations. It excluded patients with obstructing tumors because complete colonoscopy was not feasible and was required for eligibility. This requirement may have contributed to the higher number of right-sided cancers in our series compared with other reports.^8,9^ Other limitations included the retrospective nature of our study, incomplete germline genetic testing for all early-onset CRC cases, potential referral or other selection bias, and the relatively small study sample size such that validation of our study findings in a larger cohort is clearly warranted.

This study also has some strengths, including strict eligibility criteria of complete colonoscopy with adequate bowel preparation, exclusion of known hereditary syndromes or polyposis, recorded family history information, and germline genetic testing data when available. Patients were randomly selected and matched based on relevant factors of sex and indication for colonoscopy; patients with colon cancer and patients with rectal cancer were analyzed separately. Colonoscopies were performed both at Mayo Clinic sites or outside prior to referral.

## Conclusions

In our cross-sectional study, we observed a significant increase in the number of synchronous advanced adenomas distributed throughout the colon among patients with early-onset compared with average-onset colon cancer, suggesting extended field cancerization. Accordingly, patients with early-onset colon cancer are at increased risk of synchronous and metachronous CRC, and future studies that examine rates of metachronous neoplasia are needed. Among patients with early onset-rectal cancer, however, synchronous neoplasia was not increased compared with those with average-onset rectal cancer, suggesting differences in their pathobiology.

## Notes

Supplement.
Data Sharing Statement

## References

[1] Siegel RL, Miller KD, Fuchs HE, Jemal A. Cancer statistics, 2022. CA Cancer J Clin. 2022;72(1):7-33. doi:10.3322/caac.21708 35020204

[2] Bretthauer M, Løberg M, Wieszczy P, ; NordICC Study Group. Effect of colonoscopy screening on risks of colorectal cancer and related death. N Engl J Med. 2022;387(17):1547-1556. doi:10.1056/NEJMoa2208375 36214590

[3] Schoen RE, Pinsky PF, Weissfeld JL, ; PLCO Project Team. Colorectal-cancer incidence and mortality with screening flexible sigmoidoscopy. N Engl J Med. 2012;366(25):2345-2357. doi:10.1056/NEJMoa1114635 22612596 PMC3641846

[4] Zauber AG, Winawer SJ, O’Brien MJ, . Colonoscopic polypectomy and long-term prevention of colorectal-cancer deaths. N Engl J Med. 2012;366(8):687-696. doi:10.1056/NEJMoa1100370 22356322 PMC3322371

[5] Siegel RL, Miller KD, Goding Sauer A, . Colorectal cancer statistics, 2020. CA Cancer J Clin. 2020;70(3):145-164. doi:10.3322/caac.21601 32133645

[6] Zaborowski AM, Abdile A, Adamina M, ; REACCT Collaborative. Characteristics of early-onset vs late-onset colorectal cancer: a review. JAMA Surg. 2021;156(9):865-874. doi:10.1001/jamasurg.2021.2380 34190968

[7] Bailey CE, Hu CY, You YN, . Increasing disparities in the age-related incidences of colon and rectal cancers in the United States, 1975-2010. JAMA Surg. 2015;150(1):17-22. doi:10.1001/jamasurg.2014.1756 25372703 PMC4666003

[8] Cercek A, Chatila WK, Yaeger R, . A comprehensive comparison of early-onset and average-onset colorectal cancers. J Natl Cancer Inst. 2021;113(12):1683-1692. doi:10.1093/jnci/djab124 34405229 PMC8634406

[9] Chang DT, Pai RK, Rybicki LA, . Clinicopathologic and molecular features of sporadic early-onset colorectal adenocarcinoma: an adenocarcinoma with frequent signet ring cell differentiation, rectal and sigmoid involvement, and adverse morphologic features. Mod Pathol. 2012;25(8):1128-1139. doi:10.1038/modpathol.2012.61 22481281

[10] Sinicrope FA. Increasing incidence of early-onset colorectal cancer. N Engl J Med. 2022;386(16):1547-1558. doi:10.1056/NEJMra2200869 35443109

[11] Chen FW, Sundaram V, Chew TA, Ladabaum U. Advanced-stage colorectal cancer in persons younger than 50 years not associated with longer duration of symptoms or time to diagnosis. Clin Gastroenterol Hepatol. 2017;15(5):728-737. doi:10.1016/j.cgh.2016.10.038 27856366 PMC5401776

[12] Pearlman R, Frankel WL, Swanson B, ; Ohio Colorectal Cancer Prevention Initiative Study Group. Prevalence and spectrum of germline cancer susceptibility gene mutations among patients with early-onset colorectal cancer. JAMA Oncol. 2017;3(4):464-471. doi:10.1001/jamaoncol.2016.5194 27978560 PMC5564179

[13] Li S, Zhu K, Yu W, . Synchronous neoplastic lesions in referred patients with colorectal cancer: a retrospective cohort study. Cancer Manag Res. 2019;11:9951-9959. doi:10.2147/CMAR.S229376 32063721 PMC6884963

[14] Piñol V, Andreu M, Castells A, Payá A, Bessa X, Jover R; Gastrointestinal Oncology Group of the Spanish Gastroenterological Association. Synchronous colorectal neoplasms in patients with colorectal cancer: predisposing individual and familial factors. Article in Spanish. Dis Colon Rectum. 2004;47(7):1192-1200. doi:10.1007/s10350-004-0562-7 15164252

[15] Borda A, Martínez-Peñuela JM, Muñoz-Navas M, Prieto C, Betés M, Borda F. Synchronous neoplastic lesions in colorectal cancer: an analysis of possible risk factors favouring presentation. Rev Esp Enferm Dig. 2008;100(3):139-145. doi:10.1016/S0016-5085(08)62252-0 18416638

[16] Dotto GP. Multifocal epithelial tumors and field cancerization: stroma as a primary determinant. J Clin Invest. 2014;124(4):1446-1453. doi:10.1172/JCI72589 24691479 PMC3973113

[17] Hawthorn L, Lan L, Mojica W. Evidence for field effect cancerization in colorectal cancer. Genomics. 2014;103(2-3):211-221. doi:10.1016/j.ygeno.2013.11.003 24316131

[18] Fearon ER, Vogelstein B. A genetic model for colorectal tumorigenesis. Cell. 1990;61(5):759-767. doi:10.1016/0092-8674(90)90186-I 2188735

[19] Click B, Pinsky PF, Hickey T, Doroudi M, Schoen RE. Association of colonoscopy adenoma findings with long-term colorectal cancer incidence. JAMA. 2018;319(19):2021-2031. doi:10.1001/jama.2018.5809 29800214 PMC6583246

[20] Strum WB. Colorectal adenomas. N Engl J Med. 2016;374(11):1065-1075. doi:10.1056/NEJMra1513581 26981936

[21] Sweetser S, Smyrk TC, Sinicrope FA. Serrated colon polyps as precursors to colorectal cancer. Clin Gastroenterol Hepatol. 2013;11(7):760-767. doi:10.1016/j.cgh.2012.12.004 23267866 PMC3628288

[22] Corley DA, Jensen CD, Marks AR, . Variation of adenoma prevalence by age, sex, race, and colon location in a large population: implications for screening and quality programs. Clin Gastroenterol Hepatol. 2013;11(2):172-180. doi:10.1016/j.cgh.2012.09.010 22985608 PMC3954741

[23] Shaukat A, Rex DK, Shyne M, Church TR, Perdue DG. Adenoma detection rates for 45- to 49-year-old screening population. Gastroenterology. 2022;162(3):957-959. doi:10.1053/j.gastro.2021.09.028 34537208

[24] Ladabaum U, Shepard J, Mannalithara A. Adenoma and serrated lesion detection by colonoscopy indication: the ADR-ESS (ADR Extended to all Screening/Surveillance) score. Clin Gastroenterol Hepatol. 2021;19(9):1873-1882. doi:10.1016/j.cgh.2021.04.027 33895358

[25] Liang PS, Williams JL, Dominitz JA, Corley DA, Zauber AG. Age-stratified prevalence and predictors of neoplasia among U.S. adults undergoing screening colonoscopy in a national endoscopy registry. Gastroenterology. 2022;163(3):742-753. doi:10.1053/j.gastro.2022.05.036 35643172 PMC9398947

[26] Cybulski C, Wokołorczyk D, Kładny J, . Germline *CHEK2* mutations and colorectal cancer risk: different effects of a missense and truncating mutations? Eur J Hum Genet. 2007;15(2):237-241. doi:10.1038/sj.ejhg.5201734 17106448

[27] Win AK, Hopper JL, Jenkins MA. Association between monoallelic *MUTYH* mutation and colorectal cancer risk: a meta-regression analysis. Fam Cancer. 2011;10(1):1-9. doi:10.1007/s10689-010-9399-5 21061173 PMC3228836

[28] Peacock O, Vilar E, Guraieb-Trueba M, Thirumurthi S, Chang GJ, You YN. Clinically significant metachronous colorectal pathology detected among young-onset colorectal cancer survivors: implications for post-resection surveillance guidelines. Gastroenterology. 2022;163(6):1682-1684.e2. doi:10.1053/j.gastro.2022.08.030 35987446 PMC9951201

[29] Chan DS, Lau R, Aune D, . Red and processed meat and colorectal cancer incidence: meta-analysis of prospective studies. PLoS One. 2011;6(6):e20456. doi:10.1371/journal.pone.0020456 21674008 PMC3108955

[30] Gong J, Hutter C, Baron JA, . A pooled analysis of smoking and colorectal cancer: timing of exposure and interactions with environmental factors. Cancer Epidemiol Biomarkers Prev. 2012;21(11):1974-1985. doi:10.1158/1055-9965.EPI-12-0692 23001243 PMC3493822

[31] Vieira AR, Abar L, Chan DSM, . Foods and beverages and colorectal cancer risk: a systematic review and meta-analysis of cohort studies, an update of the evidence of the WCRF-AICR Continuous Update Project. Ann Oncol. 2017;28(8):1788-1802. doi:10.1093/annonc/mdx171 28407090

[32] Schumacher AJ, Chen Q, Attaluri V, McLemore EC, Chao CR. Metabolic risk factors associated with early-onset colorectal adenocarcinoma: a case-control study at Kaiser Permanente Southern California. Cancer Epidemiol Biomarkers Prev. 2021;30(10):1792-1798. doi:10.1158/1055-9965.EPI-20-1127 34301728

[33] O’Sullivan DE, Sutherland RL, Town S, . Risk factors for early-onset colorectal cancer: a systematic review and meta-analysis. Clin Gastroenterol Hepatol. 2022;20(6):1229-1240. doi:10.1016/j.cgh.2021.01.037 33524598

[34] Archambault AN, Lin Y, Jeon J, . Nongenetic determinants of risk for early-onset colorectal cancer. J Natl Cancer Inst Cancer Spectr. 2021;5(3):5. doi:10.1093/jncics/pkab029 34041438 PMC8134523

[35] Lochhead P, Chan AT, Nishihara R, . Etiologic field effect: reappraisal of the field effect concept in cancer predisposition and progression. Mod Pathol. 2015;28(1):14-29. doi:10.1038/modpathol.2014.81 24925058 PMC4265316

