# Supplementary material for: Synchronous Neoplasia Rates at Colonoscopic Diagnosis of Early-Onset vs Average-Onset Colorectal Cancer
Source: JAMA Netw Open. 2023 Jul 18;6(7):e2324038. doi: 10.1001/jamanetworkopen.2023.24038 (PMC10354682; doi:10.1001/jamanetworkopen.2023.24038)
Supplement: Supplement. — Data Sharing Statement [file jamanetwopen-e2324038-s001.pdf]

Data Sharing Statement

Emiloju. Synchronous Neoplasia Rates at Colonoscopic Diagnosis of Early-Onset vs Average-Onset Colorectal Cancer. *JAMA Netw Open*. Published July 18, 2023.
doi:10.1001/jamanetworkopen.2023.24038

Data
Data available: Yes
Data types: Deidentified participant data, Data dictionary
How to access data: Data are available upon reasonable request. sinicrope.frank@mayo.edu
When available: With publication

Supporting Documents
Document types: None

Additional Information
Who can access the data: researchers whose proposed use of the data has been approved
Types of analyses: specified purpose
Mechanisms of data availability: with a signed data access agreement
